# Calibration and validation of predicted genomic breeding values in an advanced cycle maize population

**DOI:** 10.1007/s00122-021-03880-5

**Published:** 2021-06-12

**Authors:** Hans-Jürgen Auinger, Christina Lehermeier, Daniel Gianola, Manfred Mayer, Albrecht E. Melchinger, Sofia da Silva, Carsten Knaak, Milena Ouzunova, Chris-Carolin Schön

**Affiliations:** 1 Plant Breeding, TUM School of Life Sciences, Technical University of Munich, 85354 Freising, Germany; 2 Statistical Genetics Unit, RAGT 2N, 1 Route de Moyrazès, 12510 Druelle, France; 3 Department of Animal and Dairy Sciences, University of Wisconsin-Madison, Madison, WI 53706 USA; 4 Institute of Plant Breeding, Seed Science and Population Genetics, University of Hohenheim, 70593 Stuttgart, Germany; 5 KWS SAAT SE & Co. KGaA, 37555 Einbeck, Germany

## Abstract

**Key message:**

Model training on data from all selection cycles yielded the highest prediction accuracy by attenuating specific effects of individual cycles. Expected reliability was a robust predictor of accuracies obtained with different calibration sets.

**Abstract:**

The transition from phenotypic to genome-based selection requires a profound understanding of factors that determine genomic prediction accuracy. We analysed experimental data from a commercial maize breeding programme to investigate if genomic measures can assist in identifying optimal calibration sets for model training. The data set consisted of six contiguous selection cycles comprising testcrosses of 5968 doubled haploid lines genotyped with a minimum of 12,000 SNP markers. We evaluated genomic prediction accuracies in two independent prediction sets in combination with calibration sets differing in sample size and genomic measures (effective sample size, average maximum kinship, expected reliability, number of common polymorphic SNPs and linkage phase similarity). Our results indicate that across selection cycles prediction accuracies were as high as 0.57 for grain dry matter yield and 0.76 for grain dry matter content. Including data from all selection cycles in model training yielded the best results because interactions between calibration and prediction sets as well as the effects of different testers and specific years were attenuated. Among genomic measures, the expected reliability of genomic breeding values was the best predictor of empirical accuracies obtained with different calibration sets. For grain yield, a large difference between expected and empirical reliability was observed in one prediction set. We propose to use this difference as guidance for determining the weight phenotypic data of a given selection cycle should receive in model retraining and for selection when both genomic breeding values and phenotypes are available.

**Supplementary Information:**

The online version contains supplementary material available at 10.1007/s00122-021-03880-5.

## Introduction

The prediction of breeding values from molecular data has become a key component of many plant breeding programmes. In breeding hybrid crops such as maize, genomic prediction can be applied at different stages of the breeding scheme. When beginning a new selection cycle, genome-based prediction can assist in the choice of crosses that warrant both high mean performance and high genetic variance for target traits (Lehermeier et al. 2017; Allier et al. 2019). The next step is to identify selection candidates with the highest combining ability in a large sample of testcrosses within the same heterotic group (Albrecht et al. 2011; Riedelsheimer et al. 2012; Jacobson et al. 2014) or to predict the performance of potential hybrid combinations directly (Massman et al. 2013; Technow et al. 2014; Seye et al. 2020).

For each of these prediction steps, a statistical model must be trained on experimental calibration data comprising high-quality phenotypes and genotypes. Deterministic formulas forecasting prediction accuracy suggest a strong influence of the sample size, the heritability, the genetic architecture of the target trait and the genome structure of the species under study (Daetwyler et al. 2010; Schopp et al. 2017). Simulation studies have shown that the mating design and family structure of the calibration set also have a strong influence on prediction accuracy (Hickey et al. 2014). Results from experimental studies corroborate these findings irrespective of whether the studied populations were designed for research purposes (Lehermeier et al. 2014) or originated from commercial breeding programmes (Albrecht et al. 2014; Krchov et al. 2015; Auinger et al. 2016).

In addition to the specific properties of the calibration set, the relatedness between the calibration and prediction set plays an important role (Habier et al. 2007; Saatchi et al. 2011; Clark et al. 2012; Lorenz and Nice 2017). In animal breeding, selection candidates are direct descendants of the previous selection cycle; thus, relatedness between calibration and prediction sets is given. In many plant breeding programmes, however, the situation is different. Depending on the generation interval of the crop, several years might elapse between the evaluation of parental lines and their progenies. Furthermore, plant breeders enrich the genetic diversity of advanced cycle breeding populations through crossings with unrelated or distantly related genetic material. Depending on the mating design, this practice can alter haplotype structure and linkage disequilibrium of the selection candidates dramatically compared to the population on which the prediction model was trained. While selection on phenotypes is not impaired by these proceedings, the success of genome-based selection might be jeopardised. In the worst case, the predictive power of the calibration set breaks down despite continuous retraining of the model over the years.

In breeding programmes where genome-based prediction is applied on a routine basis, a large body of data becomes available for model training. Several authors have found that prediction accuracy was impacted adversely when the calibration and the prediction sets were distantly related, and that removal of some genotypes from the calibration set improved prediction accuracy (Albrecht et al. 2014; Michel et al. 2016; Pembleton et al. 2018). Brandariz and Bernardo (2019) demonstrated that, for populations derived from biparental crosses of maize, utilising ad hoc training populations produced better results than did training the prediction model on all available data, despite a substantially smaller sample size. Their experimental data set comprised a high number of large half-sib families, and prediction accuracies were highest when the calibration set comprised families having one parent in common with the family to be predicted. In these cases, relatedness was high, and changes in linkage phase across families were negligible, as shown theoretically and empirically by Lehermeier et al. (2014). If the calibration and prediction sets do not comprise large biparental families, the creation of ad hoc calibration sets is complicated, and drivers of prediction accuracy other than sample size and relatedness are largely unknown. We, therefore, investigated several genomic measures contributing to prediction accuracy. Building on a unique data set from an advanced cycle maize breeding programme comprising high-precision phenotypic and high-density genotypic data and representing six interconnected breeding cycles, our main objectives were to (1) assess the accuracy of genomic best linear unbiased prediction (GBLUP) of different calibration sets from up to five previous selection cycles in two prediction sets, (2) investigate the impact of the genetic diversity of the calibration set, (3) examine differences between sample size and effective sample size in the calibration set and (4) investigate how variation in marker polymorphism, linkage disequilibrium and the degree of relatedness between calibration and prediction sets affect prediction accuracy.

## Materials and methods

### Plant material

The experimental data presented in this study consist of genetic material from six contiguous selection cycles of a commercial maize breeding programme (Table 1). The six data sets (S1–S6) comprise 5968 doubled haploid (DH) lines from the Dent heterotic group crossed to one or several Flint testers. Data sets were disconnected with respect to selection candidates but connected through 11 commercial check hybrids. Data sets S1 to S6 varied in size from 551 to 1545 DH lines. Individual sets were generated by crossing between 36 and 148 parents, which resulted in 130 to 607 crosses with 1 to 455 progenies per parent (Table 1). In S1 and S2, each line was crossed to one of two testers. In S3, four testers were used with 193 lines crossed to more than one tester. In S4, S5 and S6, one tester was used for each of the sets. S1 to S5 are connected by one single-cross tester, while the tester in S6 was a double cross with the common tester of S1 to S5 as a parent. Data sets S1 and S2 were part of the study of Albrecht et al. (2014). Plant materials described in this study are proprietary to KWS SAAT SE & Co. KGaA.

**Table 1:** Description of data sets S1 to S6 tested in the years 2010 to 2015, respectively. Given are the sample size (N), the number of parents and crosses from which DH lines were derived, the median [minimum–maximum] number of DH lines per parent and cross, the number of locations and the number of testers used for evaluating each data set

Data set	*N*	No. of parents	No. of lines per crosses	No. of		Locations^a^	Testers
				Parent	Cross		
S1	928	52	173	21 [1–203]	3 [1–63]	6 (4)	2
S2	842	73	287	12 [1–129]	2 [1–26]	6 (3.4)	2
S3	1085	148	246	6 [1–115]	1 [1–28]	7 (4.5)	4
S4	1017	58	130	13 [1–455]	4 [1–47]	6	1
S5	1545	145	607	5 [1–62]	2 [1–31]	5	1
S6	551	36	228	30 [2–82]	2 [1–6]	5	1

^a^If DH lines were not tested in all locations, numbers in parentheses indicate the average

### Genotypic and phenotypic data

The DH lines of sets S1 to S4 were genotyped with 56,110 SNP markers using the Illumina® MaizeSNP50 BeadChip (Ganal et al. 2011). DH lines of sets S5 and S6 were genotyped with custom-made chips comprising subsets of 12,062 and 22,359 SNPs of the Illumina® MaizeSNP50 BeadChip, respectively. Genotypic data from sets S1 to S6 were merged, and only SNPs with a GTScore ≥ 0.7 or call frequency ≥ 0.9 were retained for further analyses. Monomorphic SNPs and SNPs with the alternative allele appearing only once were removed. If two SNPs were fully collinear, one was discarded at random. In the full dataset (*S*_all_, *N* = 5968), 9742 informative SNPs remained. Missing genotypic information was imputed using the function ‘codeGeno’ from the R-package ‘synbreed’ version 0.12–14 with option ‘impute.type = “beagle”’ (Wimmer et al. 2012; Browning and Browning 2009) within the GNU-R environment (R Core Team 2020).

Data sets S1 to S6 were evaluated phenotypically in multi-location trials over 6 years (2010–2015). Within locations, testcrosses were allocated to a series of trials laid out as 10 × 10 lattices comprising additional DH lines from the same breeding programme as well as five to seven common commercial checks. The full set of genotypes was replicated twice in S1 and S2, partially in S3 and not at all in S4, S5 and S6. Testcross performance was evaluated for grain dry matter yield (GDY, dt/ha) and grain dry matter content (GDC, %). Best linear unbiased estimates (BLUEs), variance components and heritabilities within data sets S1 to S6 were estimated as described by Albrecht et al. (2014).

### Prediction of genomic breeding values

Data sets S1 to S5 served as calibration sets (CS) for model training individually and in all possible combinations resulting in 31 calibration sets ranging from *N* = 842 (S2) to *N* = 5417 (S1_2_3_4_5 = combined S1, S2, S3, S4 and S5). Data set S6 served as prediction set. Furthermore, for the 15 calibration sets involving all possible combinations of S1 to S4, data set S5 served as prediction set. In the respective calibration sets, BLUEs of DH lines averaged across locations were used as a response variable in the GBLUP model with the following form:

$${\mathbf{y}} = \mathbf{1}\mu + {\mathbf{Zu}} + {\mathbf{e}}$$

where ${\mathbf{y}}$ is a vector of the BLUEs of GDY or GDC, respectively, ***µ*** is the population mean and **u** is a vector of random genomic breeding values (GBV) with the distribution $\mathbf{u} \sim N\left(0;\mathbf{U}{\sigma }_{u}^{2}\right)$. **Z** is the corresponding incidence matrix, ${\mathbf{e}}$ is a vector of residuals, which, for simplicity, is assumed to be normally distributed with a mean of zero and equal variance ${\mathbf{e}}\sim N({0};{\mathbf{I}}\sigma^{2} ).$ The genomic kinship matrix of the genotyped DH lines ${\mathbf{U}}$ was calculated according to VanRaden (2008) with allele frequencies estimated from S_all_. Variance components ${\sigma }_{u}^{2}$ and ${\sigma }^{2}$ are the testcross and residual variances pertaining to the GBLUP model, respectively.

Prediction accuracies (*r*) were calculated using Pearson’s correlation coefficients between GBVs predicted based on the model trained with the respective calibration set and BLUEs averaged across locations of the respective prediction set, divided by the corresponding square root of the trait’s heritability. Empirical reliabilities were obtained from the squared accuracies (*r*^2^).

### Genomic measures for characterising calibration and prediction sets

The molecular diversity of data sets S1 to S6 was assessed by calculating the proportion of polymorphic markers, nucleotide diversity (Nei and Li 1979) and haplotype heterozygosity (Nei and Tajima 1981). Haplotype heterozygosity was calculated for sliding windows (Conrad et al. 2006) of 0.5 Mb, with steps of 1 SNP and a minimum number of 5 SNPs per window. If causal variants contribute to trait variation in the prediction set but are not captured by SNPs in the calibration set or vice versa, prediction accuracy is likely to deteriorate. We, therefore, calculated the parameter nPoly, which reflects the number of common polymorphic SNPs, for all 46 combinations of calibration and prediction sets (15 with S5, 31 with S6).

A principal coordinate analysis (PCoA, Gower 1966) was conducted on data set S_all_ (S1–S6, *N* = 5968 and 9742 SNPs) based on the realised kinship matrix using the R-package 'ape' version 5.3 (Paradis and Schliep 2019). Variation within and between data sets was assessed by partitioning the molecular variance of *S*_all_ in an analysis of molecular variance (AMOVA) according to Excoffier et al. (1992).

For data sets S1 to S6, the linkage disequilibrium (LD) measure ${r}^{2}$ (Hill and Robertson 1968) was calculated for pairs of SNPs located on the same chromosome, and the average LD decay distance for *r*^2^ < 0.1 was estimated using nonlinear regression (Hill and Weir 1988). We calculated linkage phase similarities (LPS) for all combinations of calibration and prediction sets according to Schopp et al. (2017):

$$\mathrm{LPS}= \frac{{\sum }_{k}^{p}{r}_{k}^{\mathrm{CS}}{r}_{k}^{\mathrm{PS}}}{\sqrt{{\sum }_{k}^{p}{\left({r}_{k}^{\mathrm{CS}}\right)}^{2}}\sqrt{{\sum }_{k}^{p}{\left({r}_{k}^{\mathrm{PS}}\right)}^{2}}}$$

where *k* is the index for the *p* marker pairs. The sign of *r*_k_^CS^ was inferred from calculating *D* = *p*_AB_ − *p*_A_*p*_B_ of marker pair *k*, where *p*_AB_ denotes the frequency of haplotype AB, *p*_A_ the frequency of allele A at one marker locus and *p*_B_ the frequency of allele B at the other locus in the calibration set.

The influence of the sample size *N* on prediction accuracy was determined by sampling at random DH lines from the combined set S1_2_3_4_5 for *N* = 100 to *N* = 5400 in increments of 100. However, in advanced cycle breeding populations, relatedness between genotypes might be highly unbalanced. Therefore, we introduced the concept of effective sample size 1 ≤ ${N}_{\mathrm{eff}}$ ≤ *N* given by the following formula:

$${N}_{\mathrm{eff}}= \frac{N}{1+\frac{N-1}{N}\mathrm{var}\left(\lambda \right)}$$

Here *N* denotes the size of the calibration set under study and *var*(*λ*) the estimated variance of the eigenvalues of the corresponding genomic kinship matrix **U**. With *N* independent genotypes, the expected variance of the eigenvalues of ${\mathbf{U}}$ is zero, and *N*_eff_ is equal to *N*. As the pattern of relatedness becomes increasingly unbalanced, one would expect var(*λ*) to increase, leading to a reduction of *N*_eff_ compared to *N*.

To assess the degree of the relatedness between the calibration and prediction sets, we adopted the approach of Saatchi et al. (2011). For all possible combinations, we calculated the average maximum realised kinship coefficient (*u*_max_) based on the genomic kinship matrix **U**. The maximum kinship of line *i* of the respective prediction set (*u*_max,*i*_) was derived as max(**U**_*ij*_) where **U**_*ij*_ are the realised kinship coefficients between line *i* and the lines *j* of the respective calibration set. Averaging over DH lines in the prediction set produced the *u*_max_ value for each combination.

For each combination of the calibration and prediction sets, the average expected reliability can be calculated from theory. Following Clark et al. (2012), we calculated the reliability of line *i* in the prediction set from its prediction error variance (PEV(*i* $\in PS\left|CS\right))$ derived from the GBLUP model employing a specific calibration set as follows:

$${\rho }^{2}\left(i \in PS|CS\right)=1-\left(\frac{PEV\left(i \in PS|CS\right)}{{\mathbf{U}}_{ii}{\sigma }_{u}^{2}}\right)$$

where ${\sigma }_{u}^{2}$ is the genomic variance pertaining to this model and **U**_*ii*_ is the diagonal element of matrix $\mathbf{U}$ referring to line *i.* By averaging over all DH lines in the prediction set, we can obtain the expected reliability estimate for each trait and each combination of the calibration and prediction sets.

To analyse the relative importance and interdependencies of sample size *N* and genomic measures ${N}_{\mathrm{eff}}$, nPoly, *u*_max_, $\mathrm{LPS}$ and trait-specific reliabilities *ρ*^2^ for differentiating the 46 combinations of the calibration and prediction sets, we conducted a principal component analysis (Jolliffe and Cadima 2016) on the centered and standardised estimates using the GNU-R environment (R Core Team 2020).

The significance of genomic measures for predicting accuracies was assessed with multiple linear regression using empirical prediction accuracies for GDY and GDC of the 46 combinations of the calibration and prediction sets as response variables, respectively. Model selection was performed using stepwise model selection in the GNU-R environment (R Core Team 2020), which involved adding and removing covariates in each step. In addition, the significance of sample size *N* and of individual genomic measures ${N}_{\mathrm{eff}}$, nPoly, *u*_max_, $\mathrm{LPS}$ and *ρ*^2^ was tested in a linear model. To account for the effect of the prediction set, a categorical covariate indicating whether prediction accuracies were estimated in S5 or S6 was included in all models.

## Results

### Phenotypic and molecular characterisation of individual data sets

Table 2 presents testcross means, variance components and heritabilities for traits GDY and GDC in data sets S1 to S6. Genotypic variance components were highly significant (*p* < 0.01) in all sets. Trait heritabilities (*h*^2^) on a progeny-mean basis were high for both traits in most data sets with the exception of GDY in S4 and S6.

**Table 2:** Mean, minimum and maximum of BLUEs, variance components and heritabilities for traits grain dry matter yield (GDY) and grain dry matter content (GDC) for data sets S1 to S6

Trait	Set	Mean	Minimum	Maximum	${\sigma }_{G}^{2}$	${\sigma }_{GxL}^{2 a}$	${h}^{2}$
	S1	128	95	146	35.6	24.3	0.85
	S2	144	111	163	43.4	41.1	0.78
GDY	S3	142	113	163	16.9	47.6	0.75
	S4	120	97	136	12.9	61.4	0.56
	S5	144	110	168	52.8	87.2	0.74
	S6	124	87	143	20.7	93.7	0.52
	S1	69	65	74	1.20	0.20	0.96
	S2	72	66	77	1.92	0.19	0.97
GDC	S3	70	66	75	0.80	2.27	0.75
	S4	69	66	73	1.04	0.53	0.92
	S5	70	67	73	0.70	0.44	0.88
	S6	69	66	72	0.88	0.61	0.88

^a^Variance component ${\sigma }_{GxL}^{2}$ represents the genotype × location and the residual variance

**Fig. 1:**
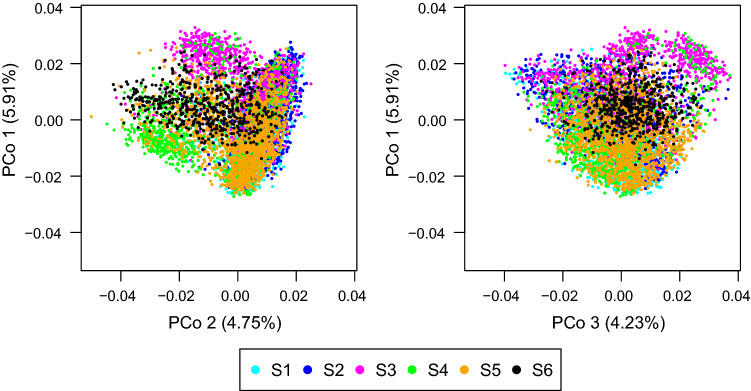
Principal coordinate analysis of pairwise realised kinship coefficients of 5968 DH lines. DH lines are coloured according to their grouping in data sets. Axis labels show the percentage of variance explained by the coordinate

The principal coordinate analysis indicated substantial overlap of the six data sets with the first three coordinates explaining 14.9% of the molecular variance (Fig. 1). In the AMOVA (Suppl. Table S1), only approximately 5% of the total molecular variance was due to variation among data sets. Within data sets S1, S3 and S4, family substructures were visible in the heatmap of pairwise realised kinship coefficients between DH lines (Suppl. Figure S1). The measure of the proportion of polymorphic markers, nucleotide diversity and haplotype heterozygosity identified S4 and S6 as the data sets with the lowest diversity (Suppl. Tables S1 and S2). Data set S6 showed twice the range of LD noted in all other data sets.

### Relatedness, reliability and linkage phase similarity of calibration and prediction sets

Table 3 and Suppl. Table S3 offer estimates of ${N}_{\mathrm{eff}}$, nPoly, *u*_max_, $\mathrm{LPS}$ and trait-specific reliabilities *ρ*^2^ calculated for all possible combinations of the calibration and prediction sets (15 for S5, 31 for S6). Data set S5 and all its combinations exhibited substantially higher ${N}_{\mathrm{eff}}$ than all other calibration sets. The number of polymorphic SNPs shared by the calibration and prediction set was higher for combinations with S5 compared to those with S6 due to the low number of polymorphic markers in data set S6. Mean estimates for *u*_max_, $LPS$ and *ρ*^2^ were similar for S5 and S6, but the range across calibration sets was larger for *u*_max_ and $LPS$ when predicting in S6. This larger range derived mainly from the low values of *u*_max_ (0.26) and $LPS$ (0.59) for the combination S1/S6 (Suppl. Table S3). Correlations between estimates of genomic measures ranged from 0.17 (${N}_{\mathrm{eff}}$, *u*_max_ for prediction in S6) to 0.93 (*u*_max_, *ρ*^2^ for prediction in S5; Table 4). Correlations of *u*_max_ with $N$ and ${N}_{\mathrm{eff}}$ differed strongly for the two prediction sets because in combinations with S6, *u*_max_ values formed two distinct groups depending on whether S3 was included or not (Suppl. Figure S2).

**Table 3:**
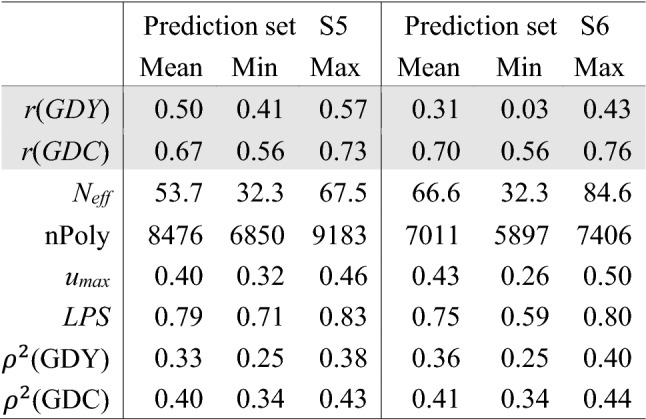
Mean and range of prediction accuracy (*r*), effective sample size (*N*_*eff*_) of calibration sets, number of polymorphic SNPs shared by the calibration and prediction set (nPoly), average maximum kinship (*u*_*max*_), linkage phase similarity (LPS) and trait-specific reliability (*ρ*^2^) for prediction sets S5 and S6 in combination with all possible calibration sets (15 for S5, 31 for S6)

**Table 4:**
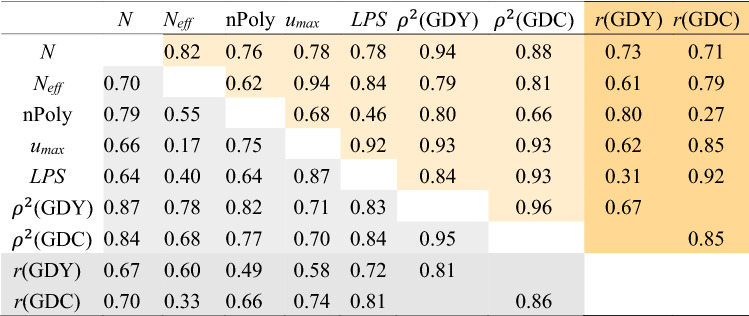
Pairwise correlations between sample size *N*, genomic measures effective sample size (*N*_eff_*)*, number of polymorphic SNPs shared by the calibration and prediction set (nPoly), average maximum kinship (*u*_max_), linkage phase similarity (LPS), expected trait-specific reliability (*ρ*^2^) and empirical trait-specific prediction accuracy (*r*). In the upper triangle, values are based on combinations of 15 calibration sets with S5 as the prediction set; in the lower triangle, values are based on combinations of 31 calibration sets with S6 as the prediction set

### Prediction accuracies

**Fig. 2:**
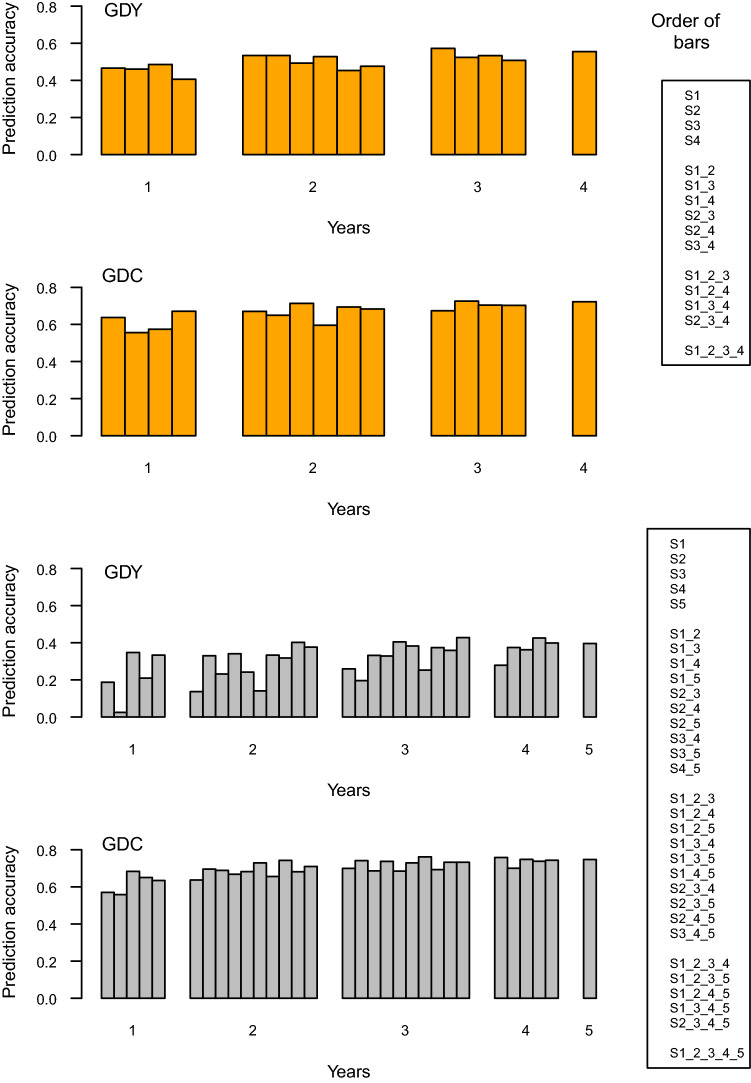
Prediction accuracies for grain dry matter yield (GDY) and grain dry matter content (GDC) for prediction set S5 (orange) and S6 (grey)

**Fig. 3:**
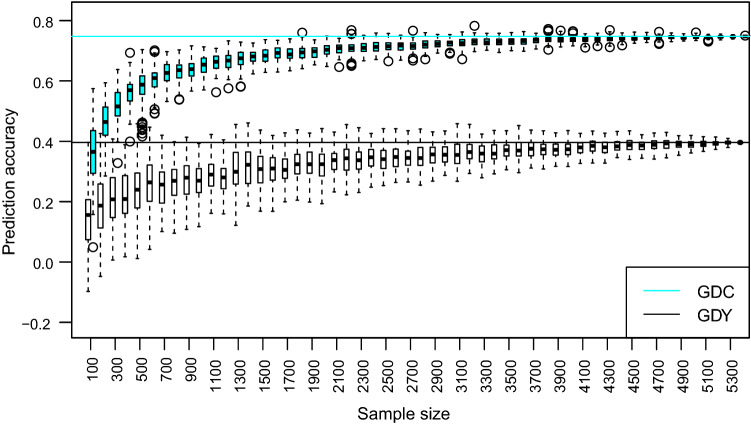
Prediction accuracy for grain dry matter yield (GDY) and grain dry matter content (GDC) as a function of sample size assessed by repeated sampling from combined calibration set S_1_2_3_4_5

Table 3 presents mean, minimum and maximum prediction accuracies in S5 and S6, and Fig. 2 and Suppl. Table S3 present prediction accuracies obtained for individual combinations of calibration and prediction sets. For GDY, the mean accuracy over all calibration sets was 0.50 for S5 and 0.31 for S6. While prediction accuracy in S5 was rather stable, varying from 0.41 (S4) to 0.57 (S1_2_3), accuracies obtained with S6 were lower and more variable (0.03 for S2 to 0.43 for S3_4_5). Compared to the accuracies for GDY, accuracies for GDC were much higher and similar for the two prediction sets. On average, prediction accuracy increased when we employed more data sets for calibration. When averaging accuracies for groups of two, three or four data sets, accuracy increased monotonically and was always highest for the combination including the largest possible number (Suppl. Table S3). Prediction accuracy increased with *N* when sampling from the combined set S1_2_3_4_5 with strongly diminishing returns for *N* > 3000 (Fig. 3). For *N* > 500, none of the samples yielded negative accuracies.

### Prediction accuracy as a function of genomic measures

**Fig. 4:**
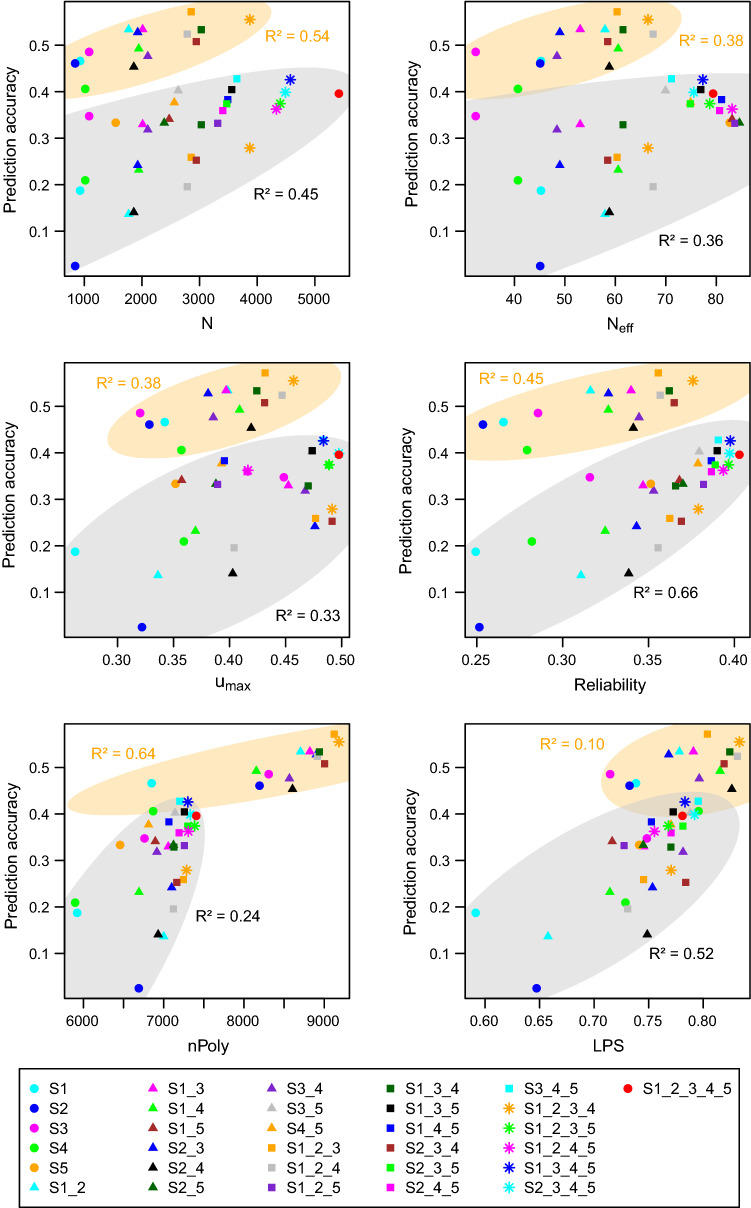
Relationship of prediction accuracy for grain yield and sample size (*N*), effective sample size (*N*_eff_), average maximum kinship (*u*_max_), reliability *ρ*^2^, number of polymorphic SNPs shared by the calibration and prediction set (nPoly) and linkage phase similarity (LPS) for 15 calibration sets predicting genomic breeding values (GBV) in S5 (orange) and 31 calibration sets predicting GBVs in S6 (grey)

The relationship of prediction accuracy for GDY with sample size $N$ and each of the genome-based measures is shown in Fig. 4 and Table 4 (for GDC in Suppl. Figures S3 and Table 4). In combination with both prediction sets, S1, S2 and S4 exhibited the lowest values for most measures and returned the lowest prediction accuracy for GDY.

To test the effect of the various measures on prediction accuracy, we fitted a linear regression model with prediction set (PS) coded as a categorical covariate. For both traits, all measures were significantly associated with prediction accuracy (Table 5). The models explained up to 80% of the variance of prediction accuracy for GDY and 75% for GDC with *ρ*^2^ as the second covariate. In the stepwise regression, only PS, nPoly and *ρ*^2^(GDY), which explained 81% of the variance of prediction accuracy for GDY (Table 5), were retained in the model. With GDC as a response variable, covariates PS, *u*_max_, ${N}_{\mathrm{eff}}$, nPoly and *ρ*^2^(GDC) were retained in the model and explained 84% of the variance.

**Table 5:** Regression analysis of prediction accuracy for grain dry matter yield (GDY) and grain dry matter content (GDC) on genomic measures characterising the 46 possible combinations of calibration and prediction sets. Significance (*p*-value), Akaike information criterion (AIC) and explained variance (*R*_adj_^2^) are given for models fitting sample size (*N*), effective sample size (*N*_eff_), number of polymorphic SNPs shared by the calibration and prediction set (nPoly), average maximum kinship (*u*_max_), linkage phase similarity (LPS) and trait-specific reliability (*ρ*^2^) in combination with the affiliation to the prediction set (PS) as covariates. The last row presents results from the best model selected by stepwise regression

GDY				GDC			
Model	*R*_adj_^2^	*p*-value	AIC	Model	*R*_adj_^2^	*p*-value	AIC
PS	0.54		− 225	PS	0.06		− 271
PS + *N*	0.74	5.9E−07	− 250	PS + *N*	0.50	1.2E−07	− 299
PS + *N*_eff_	0.70	1.4E−05	− 242	PS + ${N}_{\mathrm{eff}}$	0.23	2.5E−03	− 307
PS + nPoly	0.63	1.2E−03	− 244	PS + nPoly	0.23	2.0E−03	− 279
PS + *u*_max_	0.69	3.1E−05	− 235	PS + *u*_*max*_	0.59	2.4E−09	− 279
PS + LPS	0.73	1.7E−06	− 248	PS + LPS	0.71	8.4E−13	− 324
PS + *ρ*^2^(GDY)	0.80	1.0E−07	− 263	PS + *ρ*^2^(GDC)	0.75	4.7E−14	− 330
PS + nPoly + *ρ*^2^(GDY)	0.81		− 264	PS + *u*_max_ + ${N}_{\mathrm{eff}}$ + nPoly + *ρ*^2^(GDC)	0.84		− 347

To obtain a clearer picture of the interdependencies of the seven measures describing the 46 possible combinations of the calibration and prediction sets, we performed a principal component analysis on variables PS, sample size $N$ and the five genomic measures. Together, the first and second principal components explained 85% of the total variation (Suppl. Tables S4 and S5). Parameters $N$, *ρ*^2^ and *u*_max_ dominated the first linear component while parameters PS, nPoly and $\mathrm{LPS}$ dominated the second component.

## Discussion

The sample size of the calibration set, the relatedness of individuals in the prediction and calibration sets and the LD between markers and causal variants are major factors contributing to prediction accuracy in random mating populations (de los Campos et al. 2013). In plant breeding populations, however, the relationship between prediction accuracy and these factors is not as straightforward. Therefore, we investigated how merging data sets across several years affects prediction accuracy and related genomic measures.

### The calibration set

We analysed experimental data from a medium-sized commercial maize hybrid breeding programme. Experiments were designed for phenotypic selection. Consequently, data sets S1 to S6 were highly heterogeneous with respect to sample size, mating design, number of crosses, progenies per cross and relatedness of DH lines within and between data sets. The six data sets were connected genetically as they were derived from the same heterotic group and were adapted to the same maturity zone, but each set of DH lines experienced environmental conditions of a specific year. Because the direct progenies of crosses with selected DH lines were tested several years later (e.g. parents in S1, progenies in S4), it was important to analyse a minimum of four selection cycles. Theoretically, prediction accuracy increases with sample size, so merging as many data sets as possible for model training seems intuitively reasonable. On the other hand, it has been shown that increasing the size of the calibration set is not always beneficial (Albrecht et al. 2014; Pembleton et al. 2018; Brandariz and Bernardo 2019). Inbreeding and strong familial relationships induce cosegregation of markers and quantitative trait loci (QTL), which differ for contiguous selection cycles. Unrelated genetic material may create population substructures. Resulting phase changes between markers and QTL can have detrimental effects on prediction when the level of relatedness between the calibration and the prediction set is low (de los Campos et al. 2013).

In our study, merging data sets across years significantly increased prediction accuracy in both prediction sets and for both traits, but some calibration sets comprising only three (four) data sets yielded slightly higher accuracies than did the full set for GDY and GDC (Fig. 2, Suppl. Table S3). Thus, it might be possible to create optimised calibration sets from existing genotypic and phenotypic data. In the literature, different optimisation criteria have been suggested (Rincent et al. 2012; Isidro et al. 2015; Mangin et al. 2019; Lopez-Cruz and de los Campos 2021). However, creating bespoke calibration sets is not a simple task. A profound knowledge of the genetic makeup of the population under study is required to model systemic effects, such as genetic groups, testers, trials, locations and years, correctly (Albrecht et al. 2014). Calibration sets thus need to be optimised for each trait under study. In addition, optimisation yields the highest gains in prediction accuracy when sample sizes are small, but it is expected to show diminishing returns as the size of the calibration set increases. Considering the decreasing variation in prediction accuracy among random samples when sample size *N* increased (Fig. 3), choosing DH lines from S_all_ based on a trait-specific optimisation criterion is unlikely to be rewarding. Prediction based on the so-called general combining ability (GCA) model suggested by Brandariz and Bernardo (2019), where only crosses that share a common parent with the prediction set are used for model training, is feasible only if both the biparental families to be predicted and the breeding programme are quite large. In medium-sized breeding populations such as ours, where the number of progenies per cross and the number of crosses per parent are much lower than in their study (Table 1), the GCA model is not applicable. We, therefore, investigated if genomic measures can assist in evaluating the predictive performance of entire data sets generated in different selection cycles and tested in different years.

The results revealed that the combination of the calibration and prediction sets impacted prediction accuracies. Including S2 in model training did not negatively affect prediction accuracy in S5; when predicting GDY in S6, however, accuracies were close to zero (Fig. 2, Suppl. Table S3). Excluding S2 from model training increased prediction accuracy (e.g. prediction accuracy S1_3 > S1_2_3), despite a reduction in sample size of 842 DH lines in the calibration sets (Suppl. Figure S4). The effect of S2 was attenuated in the larger calibration sets and those including S5. Specific interactions between data sets are difficult to predict, and in fact, none of the genomic measures suggested that S2 and its combinations would exhibit such poor prediction accuracy. Thus, merging data from several selection cycles and evaluation years for model training increased the robustness of predicted GBVs in a given prediction set. When averaging accuracies over all combinations of three (four) calibration sets, predictions with the full set were always more accurate for both traits and both prediction sets (Suppl. Table S3). In addition, despite the small effective population size of this advanced cycle breeding population, prediction accuracy still increased for both traits even when sample sizes exceeded *N* = 4000.

### The prediction set

In breeding schemes with a genome-based selection step, phenotypic data collected in a given year serve several purposes. The data are used to validate GBVs of selection candidates from the previous cycle and to retrain the prediction model. Furthermore, the empirical reliabilities serve as the basis for evaluating the efficiency of genome-based selection compared to phenotypic selection and for optimising breeding schemes. A comparison of expected and empirical reliabilities in a given data set assists in evaluating the usefulness of the data.

Expected reliabilities (*ρ*^2^) were similar in magnitude and highly correlated for both prediction sets (*r* = 0.97). While empirical reliabilities for GDC were consistent with expectations, those for GDY were lower than expected, especially for the smaller calibration sets in combination with S6 (Suppl. Figure S5). Several factors might have contributed to these low empirical reliabilities. Testers in S5 and S6 were related (coefficient of coancestry = 0.5) but not identical. Empirical reliabilities are expected to decrease when a different tester is used (Schopp et al. 2015), but in a commercial maize breeding programme, correlations between testers can easily exceed 0.6, especially if testers are related (Melchinger et al. 1998). Therefore, the change in testers can partly but not fully explain the difference between expected and empirical reliability. Another factor decreasing prediction reliability might have been the specific weather conditions of the year in which data set S6 was evaluated. Two of the five locations in 2015 suffered suboptimal growing conditions, leading to low yields and location specific reliabilities close to zero (data not shown). When omitting these two locations from the analysis, the empirical reliability averaged over all calibration sets increased from 0.10 to 0.14 (Suppl. Table S6). Thus, environmental effects of the prediction year contributed to differences in expected and empirical reliabilities, but as with the change in testers, these environmental factors could not fully explain the differences.

A third factor specific to S6 was its low level of molecular and phenotypic variability. The genotypic variance component for GDY in S6 was only half that in S5, explaining the difference in heritability between the two prediction sets (Table 2). Data set S6 also shared a low number of polymorphic markers with the calibration sets and exhibited a small effective sample size (*N*_eff_ = 39.1). In addition, the range of LD blocks in S6 was approximately twice as large as in the other data sets (Suppl. Table S2). Both simulated and experimental data demonstrate that model training is ineffective when the length of haplotype blocks in the prediction set is not well represented in the calibration set (Hickey et al. 2014; Brandariz and Bernardo 2019). This might have contributed to the lower reliabilities in S6. With its high diversity, small LD blocks and large effective sample size, data set S5 was effective for both validating GBVs derived from S1 to S4 and retraining the prediction model to predict S6 (Fig. 4).

### Genomic measures

Estimates of diversity such as the proportion of polymorphic markers, nucleotide diversity or haplotype heterozygosity varied little over calibration sets S1 to S5 and did not facilitate choosing between calibration sets. With array-based data in an advanced cycle breeding population, this might have resulted from an ascertainment bias of the chip towards medium allele frequencies. All other genomic measures were highly correlated with sample size *N* and showed high mutual correlations (Table 4). Interdependencies between measures were expected; for example, the level of relatedness of a DH line with the calibration set affected *u*_max_, the reliability of its estimated breeding value and LPS. On the other hand, markers that were monomorphic in the prediction set differentiated between calibration sets with respect to relatedness but did not affect values of nPoly and LPS. To interpret dependencies between parameters, we performed a principal component analysis. The loads of the measures on the first and second component suggested two groups (Group 1: *N*, *N*_eff_, *u*_max_ and *ρ*^2^; Group 2: nPoly and LPS). Performing stepwise regression of all possible combinations of the calibration and prediction sets with prediction accuracy of GDY as the response variable confirmed this result. However, the results from the stepwise regression must be interpreted with caution because differences in Akaike information criterion (AIC) were small across the best models (Suppl. Tables S7 and S8). Only the trait-specific expected reliability *ρ*^2^ entered into all of the 10 best models for both traits. Thus, we conclude that compared with sample size *N* or *u*_max_, the average expected reliability has a higher predictive value to rank calibration sets with respect to their predictive performance in combination with a given prediction set (Table 5, Fig. 4).

The effective sample size (*N*_eff_) is a function of sample size *N* and the distribution of kinship coefficients between pairs of DH lines. Data set S5 had an extremely large effective sample size (*N*_eff_ = 82.7), which suggests a more balanced distribution of the relatedness of DH lines than in the other data sets. Because GBVs of the prediction set are weighted averages of phenotypes in the calibration set, a more uniform distribution of relatedness might result in higher prediction accuracy with an independent prediction set (de los Campos et al. 2013). The large *N*_eff_ of S5 relative to the other data sets was confirmed when drawing 1000 random samples of size *N* = 500 from each of the data sets (74.3 for S5 vs 31.2–43.5 for the others). The parameter separated S5 and all its combinations from other calibration sets (Fig. 4), but within the group containing S5, it was negatively associated with prediction accuracy for both traits (Suppl. Figure S6). Nevertheless, we conjecture that *N*_eff_ may be useful for differentiating between data sets with respect to the distribution of kinship coefficients. Its relationship with prediction accuracy warrants further research as parameter estimates vary depending on the underlying kinship matrix (**U** calculated individually for each data set vs S_all_).

We conclude that parameter *ρ*^2^ (expected reliability) is a robust predictor of the prediction accuracy obtained with different calibration sets. How much can be gained from this information in practice, though, remains to be seen. It is possible that our study has overstated the strength of association between prediction accuracies and genomic measures. Only data sets S1 to S6 are independent samples from the breeding population; because the other calibration sets consist of overlapping data sets (e.g. S1_2 and S1_3 share the DH lines of S1), they are not independent. In addition, data sets S1 and S2 had extreme values for most measures, which inflated the pairwise correlations. Nevertheless, we conjecture that strong differences between expected and empirical reliabilities of predicted GBVs are informative. The magnitude of the difference may guide decisions regarding the weight that the phenotypes of a prediction set should receive in model training for future predictions and for constructing selection indices comprising genomic breeding values and phenotypic data (Lande and Thompson 1990).

## Conclusions

Our results are relevant for integrating phenotypic and genome-based selection in hybrid breeding programmes. To obtain high prediction accuracies, the management of the population with respect to unrelated germplasm and mating design requires greater attention in genome-based than in phenotypic selection. Including data from additional cycles in model training attenuates the effects of different testers, individual years and genotype × year interactions, which represents an advantage of genome-based selection over phenotypic selection where for most crops highest selection intensities are applied to data from a single year. Because the results varied across traits and prediction sets, genomic parameters investigated in this study provided little guidance in choosing specific calibration sets for prediction. For optimal integration of phenotypic and genomic information, we, therefore, recommend including a substantial overlap of common entries between selection cycles to disentangle confounded factors contributing to the difference between expected and empirical reliabilities.

## Supplementary Information

Below is the link to the electronic supplementary material.

Supplementary file1 (PDF 77 kb)

Supplementary file2 (PDF 2069 kb)

## Data Availability

Data are proprietary to KWS SAAT SE & Co. KGaA and can be made available for research purposes in anonymous form via a material transfer agreement.
