# Supplementary material for: Calibration and validation of predicted genomic breeding values in an advanced cycle maize population
Source: Theor Appl Genet. 2021 Jun 12;134(9):3069–81. doi: 10.1007/s00122-021-03880-5 (PMC8354938; doi:10.1007/s00122-021-03880-5)
Supplement: Supplementary file 1 — Supplementary file1 (PDF 77 kb) [file 122_2021_3880_MOESM1_ESM.pdf]

Supplementary tables of

Calibration and validation of predicted genomic breeding values in an advanced cycle maize population

Hans-Jürgen Auinger, Christina Lehermeier, Daniel Gianola,
Manfred Mayer, Albrecht E. Melchinger, Sofia da Silva,
Carsten Knaak, Milena Ouzunova, Chris-Carolin Schön

Table S1: Analysis of molecular variance among and within data sets S1 to S6.

	Degrees of freedom	Variance component
Among sets	5	236
Within sets	5962	4320
Within S1	927	4350
Within S2	841	4762
Within S3	1084	4764
Within S4	1016	4015
Within S5	1544	4130
Within S6	550	3821
Total	5967	4556

Table S2: Sample size N, proportion of polymorphic markers (PP), nucleotide diversity (π), haplotype heterozygosity with window size 0.5Mb (Hhap) and the mean of chromosomewise LD decay at a level of $r^2 = 0.1$ for data sets S1 to S6.

Set	N	PP	π	Hhap	LD [Mb]
S1	928	0.923	0.223	0.490	5.93
S2	842	0.986	0.244	0.518	5.15
S3	1085	0.985	0.244	0.517	5.43
S4	1017	0.952	0.206	0.451	5.88
S5	1545	0.970	0.212	0.463	4.94
S6	551	0.766	0.196	0.438	10.28

Table S3: Effective sample size (N_{eff}) of calibration sets, number of polymorphic SNPs shared by the calibration and prediction set (nPoly) as well as average maximum kinship (u_{max}), linkage phase similarity (LPS), expected trait-specific reliability (ρ^2) and empirical trait-specific prediction accuracy (r) of (a) 15 possible calibration and prediction set combinations with S5 and (b) 31 possible calibration and prediction set combinations with S6. Minimum and maximum in bold.

(**a**) Values for prediction set S5

CS	N_{eff}	nPoly	u_{max}	LPS	ρ^2(GDY)	ρ^2(GDC)	r(GDY)	r(GDC)
S1	45.3	**6850**	0.34	0.74	0.27	0.35	0.47	0.64
S2	45.1	8194	0.33	0.73	**0.25**	**0.34**	0.46	**0.56**
S3	**32.3**	8308	**0.32**	**0.71**	0.29	0.36	0.49	0.57
S4	40.7	6869	0.36	0.80	0.28	0.38	**0.41**	0.67
Mean							0.45	0.61
S1_2	58.0	8704	0.40	0.78	0.32	0.39	0.53	0.67
S1_3	53.0	8821	0.40	0.79	0.34	0.41	0.53	0.65
S1_4	60.6	8154	0.41	0.82	0.33	0.41	0.49	0.71
S2_3	49.0	8903	0.38	0.77	0.33	0.39	0.53	0.60
S2_4	58.8	8605	0.42	0.83	0.34	0.41	0.45	0.69
S3_4	48.4	8566	0.39	0.80	0.34	0.41	0.48	0.68
Mean							0.50	0.67
S1_2_3	60.3	9124	0.43	0.80	0.36	0.41	**0.57**	0.67
S1_2_4	**67.5**	8914	0.45	0.83	0.36	0.42	0.52	**0.73**
S1_3_4	61.5	8940	0.42	0.82	0.36	0.42	0.53	0.70
S2_3_4	58.5	9004	0.43	0.82	0.36	0.42	0.51	0.70
Mean							0.53	0.70
S1_2_3_4	66.5	**9183**	**0.46**	**0.83**	**0.38**	**0.43**	0.55	0.72

(b) Values for prediction set S6

CS	N_{eff}	nPoly	u_{max}	*LPS*	ρ^2(GDY)	ρ^2(GDC)	*r*(GDY)	*r*(GDC)
S1	45.3	5922	**0.26**	**0.59**	**0.25**	**0.34**	0.19	0.57
S2	45.1	6690	0.32	0.65	0.25	0.34	**0.03**	**0.56**
S3	**32.3**	6761	0.45	0.75	0.32	0.39	0.35	0.68
S4	40.7	**5897**	0.36	0.73	0.28	0.38	0.21	0.65
S5	82.7	6457	0.35	0.74	0.35	0.39	0.33	0.63
Mean							0.22	0.62
S1_2	58.0	7001	0.34	0.66	0.31	0.38	0.14	0.64
S1_3	53.0	7057	0.45	0.75	0.35	0.41	0.33	0.70
S1_4	60.6	6692	0.37	0.71	0.32	0.42	0.23	0.69
S1_5	83.1	6895	0.36	0.72	0.37	0.41	0.34	0.67
S2_3	49.0	7100	0.48	0.75	0.34	0.40	0.24	0.68
S2_4	58.8	6931	0.40	0.75	0.34	0.41	0.14	0.73
S2_5	**84.6**	7122	0.39	0.75	0.37	0.42	0.33	0.66
S3_4	48.4	6915	0.47	0.78	0.35	0.41	0.32	0.74
S3_5	69.9	7142	0.47	0.79	0.38	0.42	0.40	0.68
S4_5	74.9	6811	0.39	0.77	0.38	0.43	0.38	0.71
Mean							0.29	0.69
S1_2_3	60.3	7246	0.48	0.75	0.36	0.42	0.26	0.70
S1_2_4	67.5	7121	0.40	0.73	0.36	0.42	0.20	0.74
S1_2_5	83.7	7258	0.39	0.73	0.38	0.43	0.33	0.69
S1_3_4	61.5	7128	0.47	0.77	0.37	0.43	0.33	0.74
S1_3_5	76.9	7258	0.47	0.77	0.39	0.43	0.40	0.68
S1_4_5	81.0	7065	0.40	0.75	0.39	0.44	0.38	0.73
S2_3_4	58.5	7164	0.49	0.78	0.37	0.42	0.25	**0.76**
S2_3_5	74.9	7300	0.49	0.78	0.39	0.43	0.37	0.69
S2_4_5	80.6	7191	0.42	0.77	0.39	0.43	0.36	0.73
S3_4_5	71.1	7201	0.48	**0.80**	0.39	0.43	**0.43**	0.73
Mean							0.33	0.72

(**b**) continued

CS	N_{eff}	nPoly	u_{max}	*LPS*	ρ^2(GDY)	ρ^2(GDC)	*r*(GDY)	*r*(GDC)
S1_2_3_4	66.5	7285	0.49	0.77	0.38	0.43	0.28	0.76
S1_2_3_5	78.7	7385	0.49	0.77	0.40	0.43	0.37	0.70
S1_2_4_5	83.1	7306	0.42	0.76	0.39	0.44	0.36	0.75
S1_3_4_5	77.3	7301	0.48	0.78	0.40	0.44	0.43	0.74
S2_3_4_5	75.5	7331	0.50	0.79	0.40	0.44	0.40	0.74
Mean							0.37	0.74
S1_2_3_4_5	79.4	**7406**	**0.50**	0.78	**0.40**	**0.44**	0.40	0.75

Table S4: Principal component analysis of affiliation to prediction set (PS), sample size (*N*), effective sample size of the calibration sets (N_{eff}), number of polymorphic SNPs shared by the calibration and prediction set (nPoly), average maximum kinship (u_{max}), linkage phase similarity (*LPS*) and reliability of grain dry matter yield (ρ^2(GDY)) assessed in 46 possible combinations of calibration and prediction sets.

	PC 1	PC 2	PC3	PC4	PC5	PC6	PC7
Portion of explained variance	0.52	0.33	0.09	0.04	0.02	0.01	0.00
Parameters							
PS	-0.15	-0.61	-0.22	-0.07	0.36	0.11	0.64
N	-0.49	-0.05	0.14	-0.51	-0.65	0.14	0.18
N_{eff}	-0.41	-0.21	0.63	0.34	0.08	-0.52	-0.00
nPoly	-0.10	0.61	0.26	-0.44	0.50	-0.07	0.33
u_{max}	-0.44	0.05	-0.63	-0.16	0.13	-0.54	-0.27
LPS	-0.33	0.45	-0.25	0.63	-0.18	0.16	0.42
ρ^2(GDY)	-0.51	-0.06	0.08	0.06	0.37	0.62	-0.46

Table S5: Principal component analysis of affiliation to prediction set (PS), sample size (N), effective sample size of the calibration sets (N_{eff}), number of polymorphic SNPs shared by the calibration and prediction set (nPoly), average maximum kinship (u_{max}), linkage phase similarity (LPS) and reliability of grain dry matter yield (ρ^2(GDC)) assessed in 46 possible combinations of calibration and prediction sets.

	PC 1	PC 2	PC3	PC4	PC5	PC6	PC7
Portion of explained variance	0.51	0.33	0.09	0.04	0.01	0.01	0.00
Parameters							
PS	-0.13	-0.62	-0.21	0.07	0.26	-0.31	0.62
N	-0.49	-0.08	0.17	0.54	-0.29	0.55	0.22
N_{eff}	-0.39	-0.23	0.66	-0.28	-0.30	-0.42	-0.14
nPoly	-0.12	0.60	0.26	0.43	0.34	-0.44	0.26
u_{max}	-0.45	0.03	-0.61	0.23	-0.24	-0.40	-0.39
LPS	-0.35	0.43	-0.24	-0.59	-0.21	0.10	0.49
ρ^2(GDC)	-0.51	-0.05	0.02	-0.22	0.73	0.26	-0.30

Table S6: Predictive ability (PA) and prediction accuracy (*r*) for grain dry matter yield (GDY) and grain dry matter content (GDC) in S6 based on BLUEs averaged over all locations and best performing locations only.

	PA(GDY)		*r*(GDY)		PA(GDC)		*r*(GDC)	
	All	Best	All	Best	All	Best	All	Best
Set	locations		locations		locations		locations	
S1	0.14	0.17	0.19	0.22	0.53	0.56	0.57	0.59
S2	0.02	0.10	0.03	0.14	0.52	0.55	0.56	0.58
S3	0.25	0.28	0.35	0.38	0.64	0.65	0.68	0.68
S4	0.15	0.19	0.21	0.26	0.61	0.59	0.65	0.62
S5	0.24	0.29	0.33	0.39	0.60	0.61	0.63	0.64
S1_2	0.10	0.18	0.14	0.24	0.60	0.61	0.64	0.64
S1_3	0.24	0.28	0.33	0.38	0.65	0.66	0.70	0.69
S1_4	0.17	0.19	0.23	0.26	0.65	0.64	0.69	0.68
S1_5	0.25	0.30	0.34	0.40	0.63	0.64	0.67	0.68
S2_3	0.17	0.23	0.24	0.32	0.64	0.65	0.68	0.68
S2_4	0.10	0.18	0.14	0.24	0.68	0.68	0.73	0.72
S2_5	0.24	0.29	0.33	0.39	0.62	0.64	0.66	0.67
S3_4	0.23	0.26	0.32	0.35	0.70	0.69	0.74	0.73
S3_5	0.29	0.31	0.40	0.42	0.64	0.65	0.68	0.69
S4_5	0.27	0.32	0.38	0.43	0.67	0.67	0.71	0.71
S1_2_3	0.19	0.25	0.26	0.34	0.66	0.66	0.70	0.70
S1_2_4	0.14	0.21	0.20	0.28	0.70	0.70	0.74	0.73
S1_2_5	0.24	0.30	0.33	0.40	0.64	0.66	0.69	0.70
S1_3_4	0.24	0.28	0.33	0.37	0.69	0.69	0.74	0.72
S1_3_5	0.29	0.32	0.40	0.43	0.64	0.66	0.68	0.69
S1_4_5	0.28	0.32	0.38	0.43	0.68	0.69	0.73	0.73
S2_3_4	0.18	0.24	0.25	0.32	0.71	0.71	0.76	0.75
S2_3_5	0.27	0.30	0.37	0.40	0.65	0.66	0.69	0.70
S2_4_5	0.26	0.32	0.36	0.42	0.69	0.70	0.73	0.74
S3_4_5	0.31	0.33	0.43	0.45	0.69	0.69	0.73	0.73
S1_2_3_4	0.20	0.26	0.28	0.35	0.71	0.71	0.76	0.75
S1_2_3_5	0.27	0.31	0.37	0.42	0.66	0.67	0.70	0.71
S1_2_4_5	0.26	0.32	0.36	0.43	0.70	0.71	0.75	0.75
S1_3_4_5	0.31	0.34	0.43	0.46	0.69	0.70	0.74	0.74
S2_3_4_5	0.29	0.32	0.40	0.44	0.70	0.71	0.74	0.74
S1_2_3_4_5	0.29	0.33	0.40	0.44	0.70	0.71	0.75	0.75
Average	0.22	0.27	0.31	0.36	0.65	0.66	0.70	0.70

Table S7: Ten best models identified by stepwise regression for trait grain dry matter yield. Models include different subsets of parameters, affiliation to prediction set (PS), sample size (N), effective sample size of the calibration sets (N_{eff}), number of polymorphic SNPs shared by the calibration and prediction set (nPoly), average maximum kinship (u_{max}), linkage phase similarity (LPS) and expected reliability of grain dry matter yield (ρ^2), Akaike information criterion (AIC), p-value, adjusted R^2 (R_{adj}) and R^2 for the 10 best models identified by stepwise regression for trait grain dry matter yield.

Model	AIC	p-value	R^2_{adj}	R^2
PS+nPoly+ρ^2	-264.71	6.66E-16	0.81	0.82
PS+ρ^2	-263.51	2.22E-16	0.80	0.81
PS+u_{max}+nPoly+ρ^2	-262.84	5.77E-15	0.81	0.82
PS+nPoly+LPS+ρ^2	-262.82	5.77E-15	0.81	0.82
PS+N_{eff}+nPoly+ρ^2	-262.75	6.00E-15	0.81	0.82
PS+N+nPoly+ρ^2	-262.72	6.00E-15	0.81	0.82
PS+u_{max}+nPoly+LPS+ρ^2	-261.74	2.91E-14	0.81	0.83
PS+LPS+ρ^2	-261.57	2.89E-15	0.80	0.81
PS+N_{eff}+ρ^2	-261.54	2.89E-15	0.80	0.81
PS+u_{max}+ρ^2	-261.52	2.89E-15	0.80	0.81

Table S8: Ten best models identified by stepwise regression for trait grain dry matter content. Models include different subsets of parameters, affiliation to prediction set (PS), sample size (N), effective sample size of the calibration sets (N_{eff}), number of polymorphic SNPs shared by the calibration and prediction set (nPoly), average maximum kinship (u_{max}), linkage phase similarity (LPS) and expected reliability of grain dry matter content (ρ^2), Akaike information criterion (AIC), p-value, adjusted R^2 (R_{adj}) and R^2 for the 10 best models identified by stepwise regression for trait grain dry matter content.

Models	AIC	p-value	R^2_{adj}	R^2
PS+u_{max}+N_{eff}+nPoly+ρ^2	-347.75	1.11E-15	0.84	0.85
PS+N+u_{max}+N_{eff}+nPoly+ρ^2	-346.13	7.33E-15	0.83	0.85
PS+u_{max}+N_{eff}+nPoly+LPS+ρ^2	-345.75	8.55E-15	0.83	0.85
PS+N+N_{eff}+nPoly+LPS+ρ^2	-345.68	8.88E-15	0.83	0.85
PS+u_{max}+nPoly+ρ^2	-345.54	8.88E-16	0.82	0.84
PS+N_{eff}+nPoly+LPS+ρ^2	-345.32	3.33E-15	0.83	0.85
PS+N+N_{eff}+nPoly+ρ^2	-344.76	4.22E-15	0.82	0.84
PS+N_{eff}+nPoly+ρ^2	-344.74	1.33E-15	0.82	0.84
PS+N+u_{max}+N_{eff}+nPoly+LPS+ρ^2	-344.24	4.55E-14	0.83	0.86
PS+N+u_{max}+nPoly+ρ^2	-343.76	6.55E-15	0.82	0.84
