# Supplementary material for: Calibration and validation of predicted genomic breeding values in an advanced cycle maize population
Source: Theor Appl Genet. 2021 Jun 12;134(9):3069–81. doi: 10.1007/s00122-021-03880-5 (PMC8354938; doi:10.1007/s00122-021-03880-5)
Supplement: Supplementary file 2 — Supplementary file2 (PDF 2069 kb) [file 122_2021_3880_MOESM2_ESM.pdf]

Supplementary figures of

Calibration and validation of predicted genomic breeding values in an advanced cycle maize population

Hans-Jürgen Auinger, Christina Lehermeier, Daniel Gianola,
Manfred Mayer, Albrecht E. Melchinger, Sofia da Silva,
Carsten Knaak, Milena Ouzunova, Chris-Carolin Schön

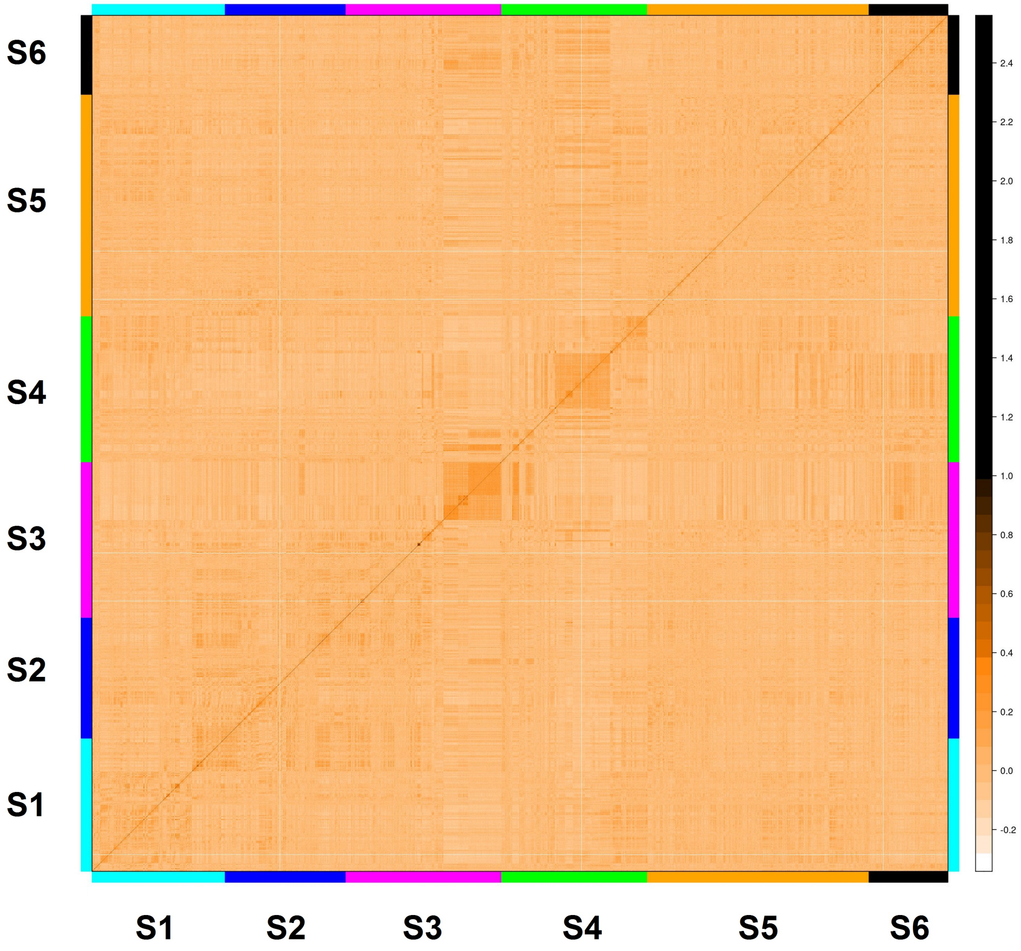

Figure S1: Heatmap of pairwise kinship coefficients in data set S_{all} (N = 5,968, M = 9,742). DH lines were hierarchically clustered within individual data sets using the unweighted pair group method with arithmetic mean.

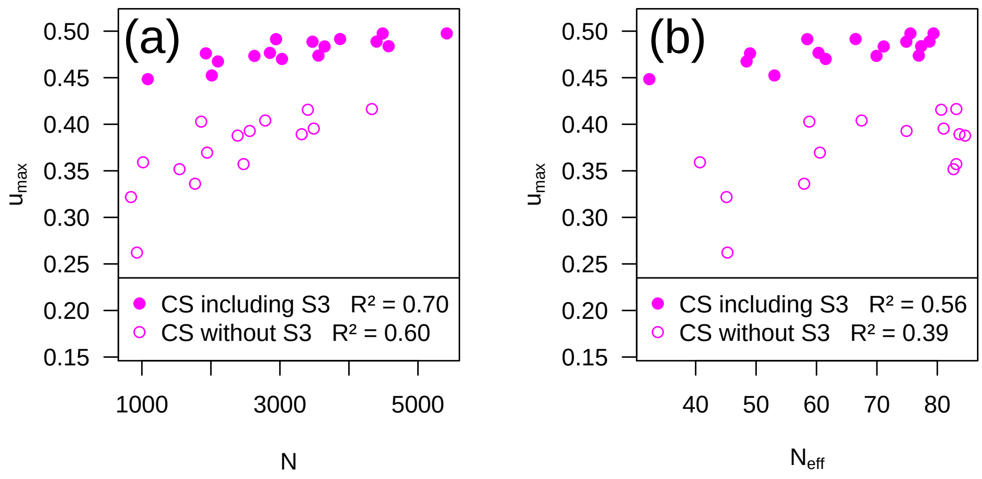

Figure S2: Relationship of (a) size of the calibration set (N) and (b) effective sample size of the calibration set (N_{eff}) with average maximum kinship (u_{max}) in combination with prediction set S6 for 16 calibration sets including data set S3 and 15 calibration sets not including data set S3.

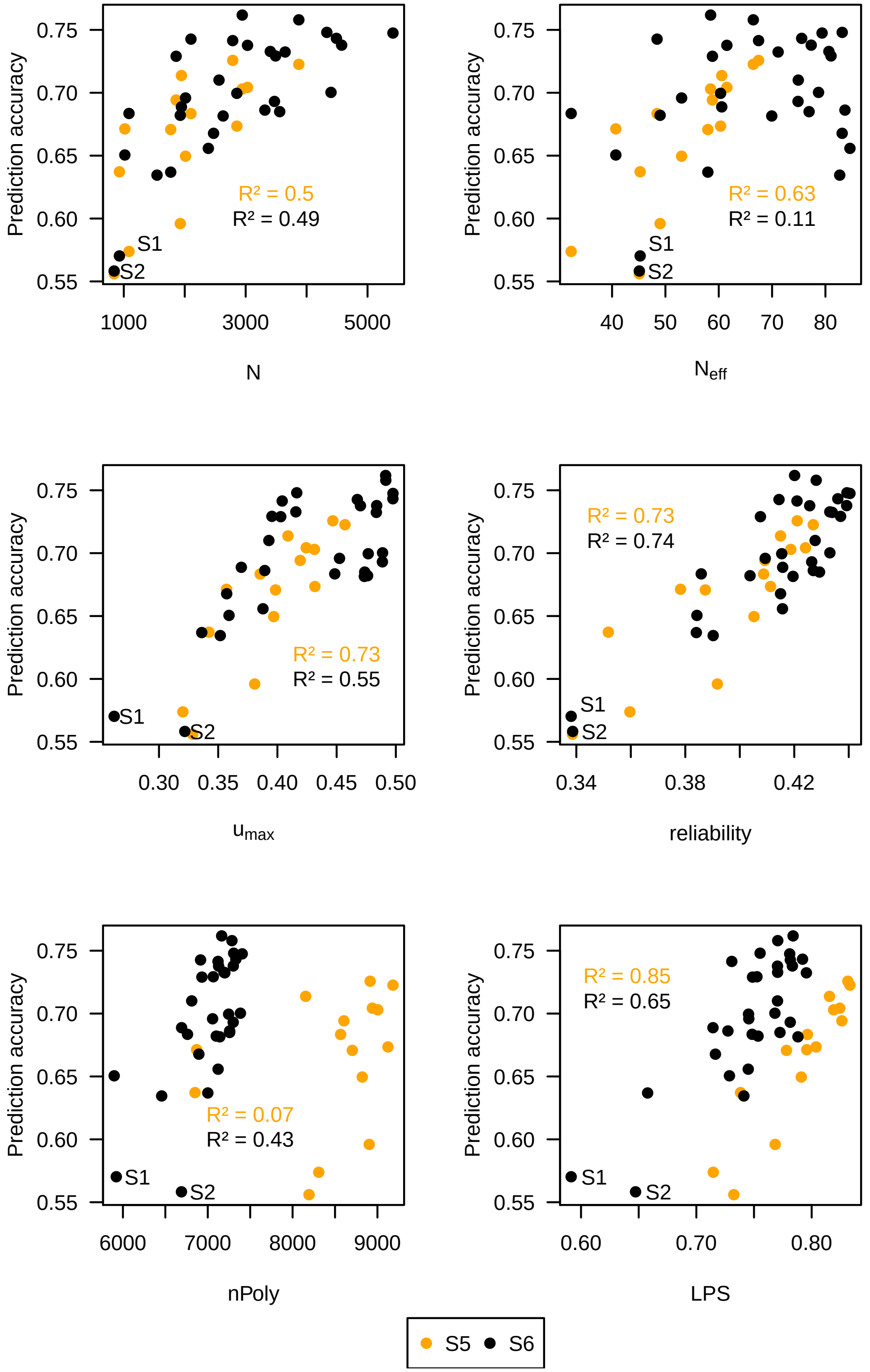

Figure S3: Relationship of prediction accuracy for grain dry matter content and the parameters sample size (N), effective sample size (N_{eff}), number of polymorphic SNPs shared by the calibration and the prediction set (nPoly), average maximum kinship (u_{max}), linkage phase similarity (LPS), and the expected reliability for 15 calibration sets predicting genomic breeding values (GBV) in S5 and 31 calibration sets predicting GBVs in S6.

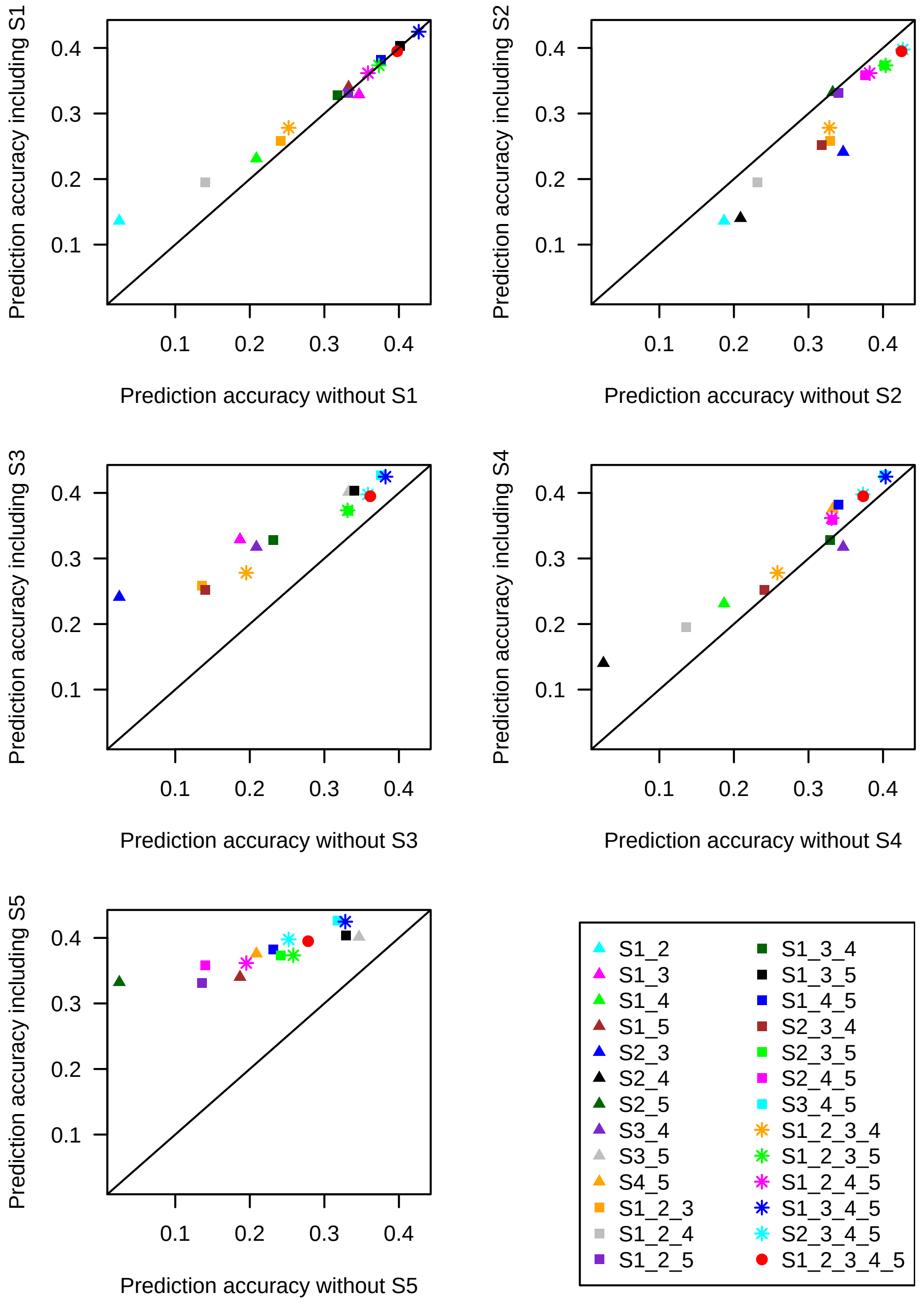

Figure S4: Relationship of prediction accuracies for grain dry matter yield in S6 obtained with calibration sets including a specific data set (e.g. all possible calibration sets including S1) and corresponding accuracies obtained with calibration sets not including the specific set. Colour coding refers to the combinations including the specific set.

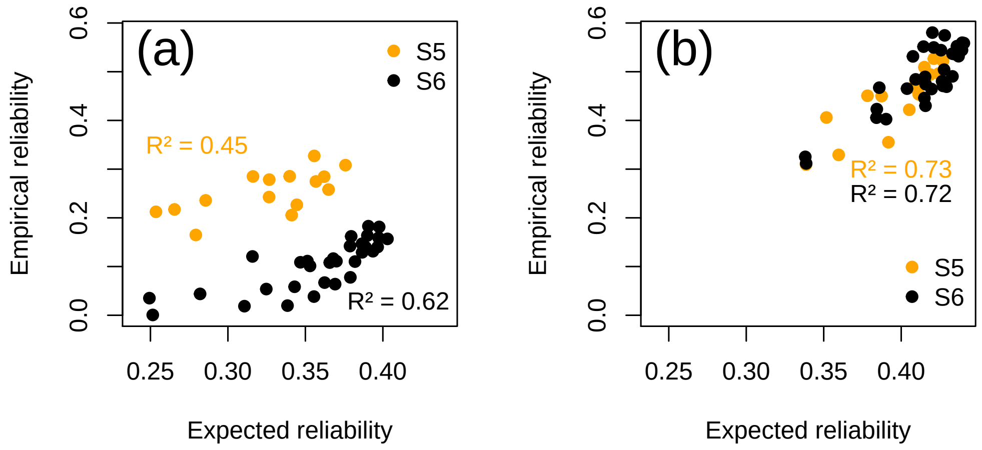

Figure S5: Relationship of empirical reliability and expected reliability for (a) grain dry matter yield and (b) grain dry matter content for 15 calibration sets predicting genomic breeding values in S5 and 31 calibration sets predicting genomic breeding values in S6.

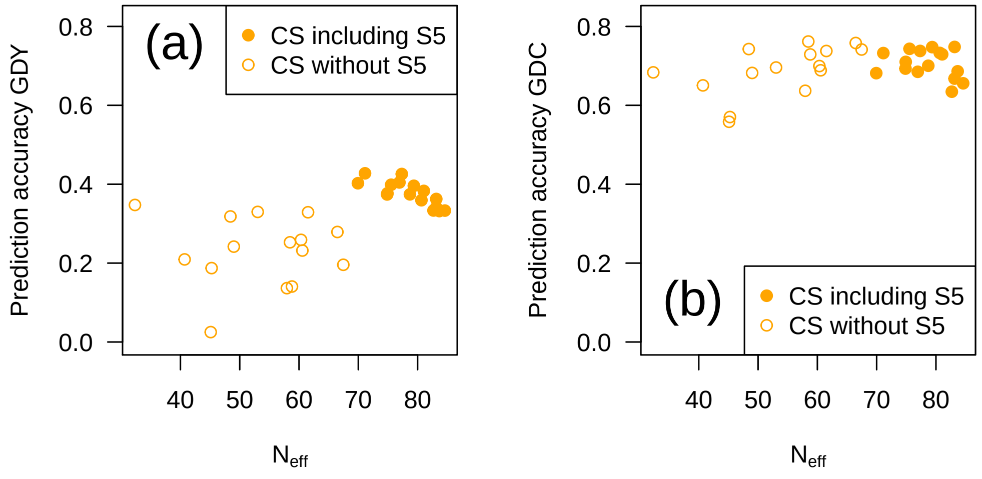

Figure S6: Relationship of effective sample size of the calibration set (N_{eff}) for 16 calibration sets including data set S5 and 15 calibration sets not including data set S5 with prediction accuracy in S6 in (a) for grain dry matter yield (GDY) and (b) for grain dry matter content (GDC).
